# On Roth’s “human fossil” from Baradero, Buenos Aires Province, Argentina: morphological and genetic analysis

**DOI:** 10.1186/s13358-023-00293-3

**Published:** 2023-10-04

**Authors:** Lumila Paula Menéndez, Chiara Barbieri, Idalia Guadalupe López Cruz, Thomas Schmelzle, Abagail Breidenstein, Rodrigo Barquera, Guido Borzi, Verena J. Schuenemann, Marcelo R. Sánchez-Villagra

**Affiliations:** 1 Department for the Anthropology of the Americas, University of Bonn, Bonn, Germany; 2 Department of Evolutionary Biology, University of Vienna, Vienna, Austria; 3 Department of Evolutionary Biology and Environmental Studies, University of Zurich, Zurich, Switzerland; 4 Escuela Nacional de Antropología e Historia, Mexico City, Mexico; 5 Department of Paleontology, University of Zurich, Zurich, Switzerland; 6 Institute of Evolutionary Medicine, University of Zurich, Zurich, Switzerland; 7 Department of Anthropology, Binghamton University, Binghamton, USA; 8 Department of Archaeogenetics, Max Planck Institute for Evolutionary Anthropology, Leipzig, Germany; 9 Centro de Investigaciones Geológicas, La Plata, Argentina; 10 Facultad de Ciencias Naturales y Museo, Universidad Nacional de La Plata, La Plata, Argentina; 11 Centro de Investigaciones Geológicas, CONICET-UNLP, La Plata, Argentina

**Keywords:** Pampas, Holocene, Anthropology collections, ancient DNA, 3D virtual reconstruction

## Abstract

The “human fossil” from Baradero, Buenos Aires Province, Argentina, is a collection of skeleton parts first recovered by the paleontologist Santiago Roth and further studied by the anthropologist Rudolf Martin. By the end of the nineteenth century and beginning of the twentieth century it was considered one of the oldest human skeletons from South America's southern cone. Here, we present the results of an interdisciplinary approach to study and contextualize the ancient individual remains. We discuss the context of the finding by first compiling the available evidence associated with the historical information and any previous scientific publications on this individual. Then, we conducted an osteobiographical assessment, by which we evaluated the sex, age, and overall preservation of the skeleton based on morphological features. To obtain a 3D virtual reconstruction of the skull, we performed high resolution CT-scans on selected skull fragments and the mandible. This was followed by the extraction of bone tissue and tooth samples for radiocarbon and genetic analyses, which brought only limited results due to poor preservation and possible contamination. We estimate that the individual from Baradero is a middle-aged adult male. We conclude that the revision of foundational collections with current methodological tools brings new insights and clarifies long held assumptions on the significance of samples that were recovered when archaeology was not yet professionalized.

## Introduction: the historical relevance of the “Baradero skeleton” in South American anthropological studies

With the professionalization of archaeology and the study of the human past, the last two centuries have seen a heated debate regarding when and how humans arrived in the Americas (Bennett et al., 2021; Boëda et al., 2014; Holen et al., 2018; Goebel et al., 2008; Hrdlička, 1912; Lund, 1842; Meltzer, 2021). The debate was first prompted by nineteenth century naturalists and travelers who were searching for fossils representing the most ancient humans and still continues today with new contributions of specialists from various fields of biological anthropology and paleogenetics working in close collaboration with archaeologists (Gonzalez-José et al., 2008; Pucciarelli et al., 2010; Ramallo et al., 2013; Scott et al., 2016). As a result, there is currently a large amount of data and publications on the topic, including recent reviews published as articles and books (De la Fuente et al., 2021; Meltzer, 2021; Nägele et al., 2022; Raff, 2022; Strauss, 2022; Willerslev & Meltzer, 2021). However, most of the proposed models or tested hypotheses were based on the repeated analyses of the same few individuals originating from only a handful of archaeological sites. Moreover, the combination of data from different sources has become a considerable challenge that this research field is currently facing (Menéndez et al., 2022).

One of the most substantial sources of evidence for discussing human history in the Americas is the ancient human skeletons themselves. Their investigation provides direct information on many aspects of the ancient past including an individuals’ lifestyle, sex and age, diet, health and disease, as well as on the evolutionary history of the populations to which these individuals belong (Brothwell, 1981; Hirst et al., 2023; Larsen, 2015). However, bone preservation is contingent and strongly depends on the environmental and cultural conditions of the burial context. As a result, the deeper we dig into the past, the less probable it is to find ancient skeletons, with an increasing decay of viable molecules to conduct chronological, isotopic, and/or genetic analyses. Particularly, skeletons from the late Pleistocene to middle Holocene are scarce in the Americas, likely due to several factors including poor archaeological visibility, bad preservation, or mortuary behaviors which do not preserve the bodies (e.g., cremation) (Dillehay, 1997). Even when skeletal remains are found, the probability of finding viable DNA and having enough collagen to be preserved for conducting biomolecular analyses is very low. Since there are very few ancient human skeletons, their study should be approached with an interdisciplinary perspective by prioritizing different steps for retrieving samples while taking an integral, respectful, and sensitive approach towards them and the related indigenous communities (Ávila-Arcos et al., 2022; Claw et al., 2018; Kowal et al., 2023; Menéndez et al., 2022).

The human skeleton from Baradero has been part of the evidence that was once studied when discussing the antiquity of humans in the Americas. It was originally recovered from the field in 1887 by the Swiss paleontologist Santiago Roth, who described the finding as a “human fossil” from the Tertiary Pampean layers (Roth, 1888, 1889). It was later mentioned and studied by Robert Lehmann-Nitsche (1907), Rudolf Martin (1907), Ales Hrdlička (1912), and Alfredo Castellano (1924). While Lehmann-Nitsche and Martin described this skeleton as the oldest human fossil from the Americas (Lehmann-Nitsche, 1907; Martin, 1907), Hrdlička argued that the skeleton instead belonged to the Quaternary and not the Tertiary, based on assessments made by Carl Burckhardt when visiting the site (Hrdlička, 1912). Castellano used the measurements published by Martin (1907) to compare it to other “human fossils” from the Southern Cone (Castellano, 1924). After this, the skeletal remains of the Baradero individual were rarely mentioned in biological anthropology or archaeology literature, and scarcely studied in the following decades. Moreover, this presumably ancient individual received less attention compared to other remains from South America. Other, more studied individuals include the Sumidouro series recovered by Peter Lund, the “Diprothomo” and “Tetraprothomo” specimens described by Florentino Ameghino, and the Fontezuelas (or Pontimelo) skeleton, which was also recovered by Santiago Roth (Sánchez-Villagra et al., 2023); a few of which become popular in the late 19^th^ and early twentieth century among scholars interested in anthropology and archaeology (Ameghino, 1880, 1887, 1909; Hrdlička, 1912; Lund, 1845; Mochi, 1910; Roth, 1889; Schwalbe, 1910).

In this paper, we present the results of an interdisciplinary project aimed at studying the Baradero individual. We first compiled all available evidence associated with the historical information together with the scientific publications on this individual to outline the historical context of the finding. Then, we conducted an osteobiographical assessment of the skeleton, by which we evaluated the sex, age, and overall conservation of the remains, comparing our results with those that were previously published. We then selected the skull fragments and mandible for conducting micro-CT-scans, while postcranial elements were surfaced-scanned. This was followed by the extraction of bone tissue and tooth samples for conducting radiocarbon and genetic analyses. Finally, we conducted a virtual reconstruction of the skull using the independent skull fragments that were micro-CT-scanned.

## Archaeological context of the finding and the history of the study of the “Baradero skeleton”

### The finding of the Baradero skeleton

**Fig. 1:**
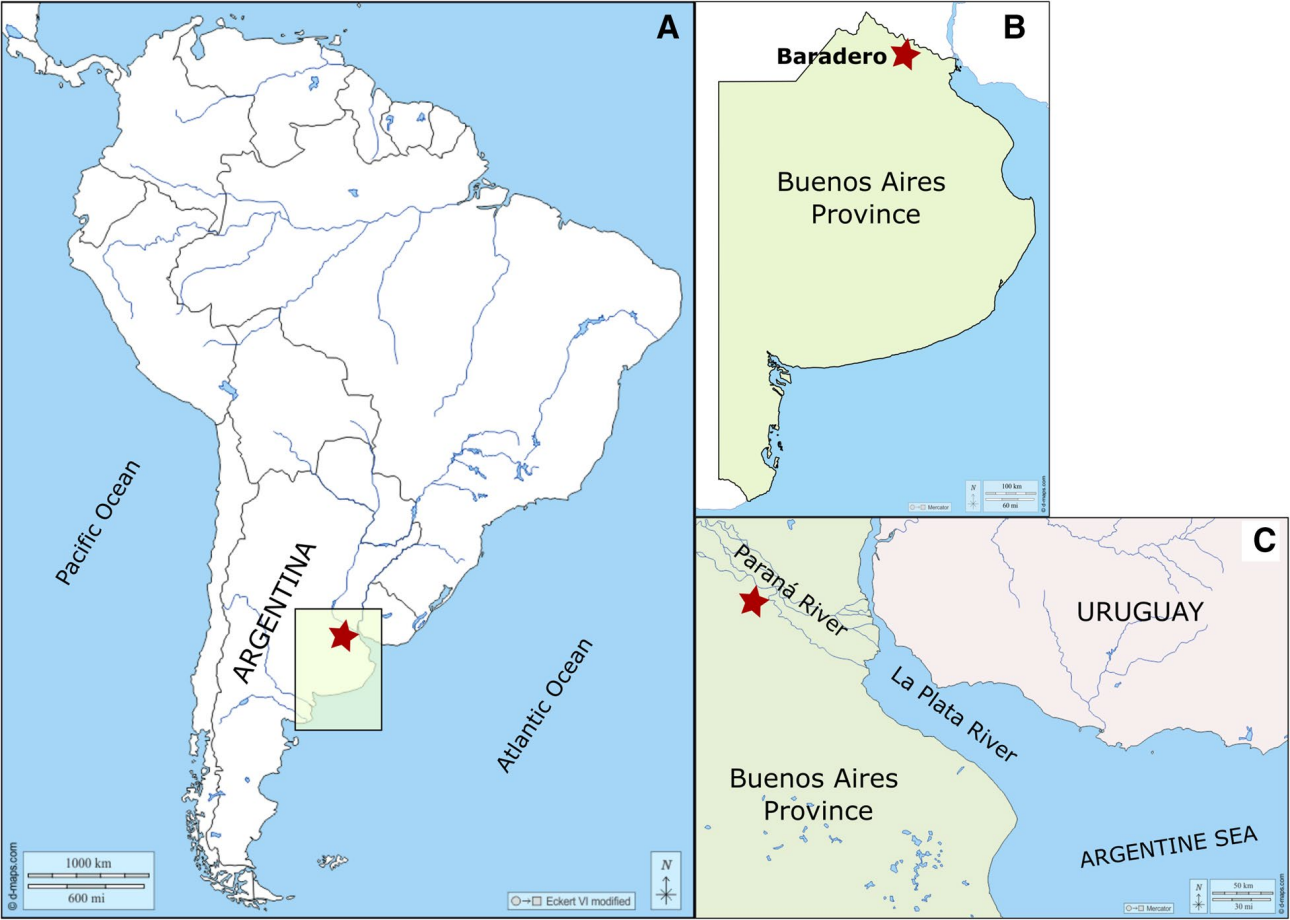
Map of South America (**a**) showing the location of Buenos Aires Province (**b**) and the approximate location of the site “Rincón del Baradero”, where the Baradero skeleton was found (indicated with a star) next to the Paraná River (**c**)

The Baradero skeleton was found in 1887 by workers in a trench that was opened for the construction of railroad tracks from the “Ferrocarril General Bartolomé Mitre” connecting the cities of Zárate and San Pedro in the north of Buenos Aires Province, Argentina (Fig. 1; Scheinsohn, 2021). As the finding was very close to the Swiss colony of Baradero, where the Swiss-Argentinean paleontologist Santiago Roth lived, he was immediately given notice of the discovery (Scheinsohn, 2021).

**Fig. 2:**
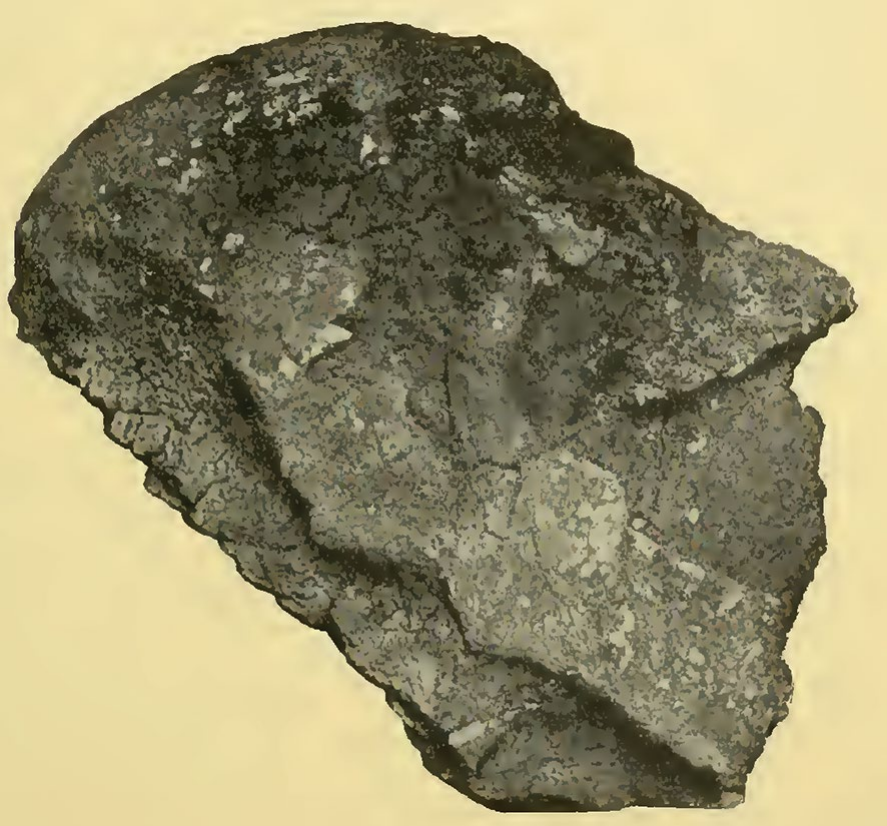
Picture probably taken by Santiago Roth showing the finding of the Baradero skull. While the resolution of the picture is low, we can still distinguish the maxilla (right upper quadrant) and the mandible (right lower quadrant) likely articulated to each other (Original source: Title “Fig. 48”; Author Lehmann-Nitsche, 1907: 377; URL: https://publicaciones.fcnym.unlp.edu.ar/rmlp/article/view/1246; License: CC BY 4.0.)

Santiago Roth (1850–1924), born as Kaspar Jacob Roth in Herisau, Switzerland, was a fossil aficionado who migrated with his family to Argentina in 1866 and settled in the Swiss colony in Baradero, the oldest town of Buenos Aires Province, next to the Paraná River (Sánchez-Villagra et al., 2023). From a young age, Roth was interested in the natural sciences, and as soon as he arrived in Baradero, he started collecting paleontological specimens. This activity shaped his professional life, since he became an active fossil finder and international fossil dealer who contributed to set the foundations of paleontology in Argentina (Sánchez-Villagra et al., 2023). As a result, he became a mediator between Argentina and Europe in the acquisition of fossil samples for natural history museums (Scheinsohn, 2021). In 1880, and as a result of selling a fossil collection to the Geneva Museum, he connected with Carl Vogt who initiated him into systematic geology and paleontology analyses (Ricciardi, 2000). Later, in 1895, Francisco P. Moreno offered him the direction of the Paleontology Department at the prestigious (and by then world-famous) Museo de La Plata, a position that he held until his last days (Bond, 1999; Farro, 2008). During his life, he made two relevant findings for biological anthropology at sites located in northern Buenos Aires Province. In 1881 he found a human skeleton that is nowadays known as the Fontezuelas skeleton (also known as the Pontimelo skeleton). This skeleton, which was found half-buried within the shell of a Glyptodon (Roth, 1888, 1889), is today housed at the Zoology Museum in Copenhagen, Denmark, and it has been dated to the late Holocene times despite presenting morphological affinities to other early Holocene individuals from the Argentinean Pampas (1985 ± 15 ^14^C years BP; Politis & Bonomo, 2011; Menéndez et al., 2015). Later, in 1887, Roth recovered the “Baradero skeleton”, probably in its original stratigraphic context, as the illustration from the schematic geological profile shows (Fig. 2; Lehmann-Nitsche, 1907).

At the time the Baradero skeleton was found, Roth was very familiar with walking the Argentinean Pampean fields searching for fossils that he could sell to museums abroad (Podgorny, 2001). There is no detailed information documenting the context of the finding and he never published a full description or any account on the skeleton. The only information available from the time is a picture that was probably taken by Roth and a letter that Roth wrote to Prof. Johannes Kollmann in the summer of 1889 (Roth, 1889). In that letter, he mentions that the skeleton was recovered from a site located two kilometers from the Baradero train station, near the swamp between the cities of San Pedro and Baradero. He describes the circumstances of the finding in such a way that he first recognized the foot bones, which were exposed in the stratigraphic profile, and then decided to proceed with the excavation of the whole skeleton (Roth, 1889). The picture taken by Roth was later published by Prof. Rudolf Martin in his report on this individual (Fig. 2; Martin, 1907) as part of the monograph written by Lehmann-Nitsche (1907) on the “Pampean Formation” and the human skeletons associated with those geological layers. It shows the skull as it was found and extracted possibly within a matrix of sediment that was keeping the cranial bones together (Fig. 2).

### Early studies on the Baradero skeleton: the information provided by Roth

Based on the context of the finding, Roth made some interpretations of the burial behavior and taphonomy. First, he considered that it was a burial in a “normal position” (Roth, 1889); meaning it was a primary burial (i.e., a burial in which the bones of the skeleton are articulated and in anatomic position). He also described the skull as being tilted forward on the chest with the vault pointing upwards, the lower jaw wide open, and the upper limbs as being very close to the knee joint (Roth, 1889). The latter could be interpreted as the result of the individual being buried in a foetal position. Although not mentioned by him, we believe that this positioning could be the result of post-depositional processes. Second, he describes the poor preservation of the bones and interprets this as the result of the skeleton lying on the surface for a very long time and having been exposed to the wind and rain (Roth, 1889), i.e., without being intentionally buried but rather the individual being alone or left behind at the moment of death. Martin disagreed with this idea and instead considered that the skeleton had been deeply buried and also subjected to a powerful pressure that fragmented most of its parts (Martin, 1907). Finally, Roth considered this skeleton to be ancient not only due to it being found within the stratigraphic loess layer that he defined as “Pampeano Intermedio” (“Intermediate Pampean”, which for him corresponded to Pliocene times; Voglino et al., 2023) but also because of its particular skull shape that he considered as being “primitive” (Roth, 1888, 1889). In agreement with this, Martin acknowledged in his report that this was the most ancient human skeleton of Argentina, although he commented that the morphology was that of a contemporary human and not a predecessor species (Martin, 1907).

### Further studies on the Baradero skeleton: insights from Burckhardt, Lehmann-Nitsche, and Martin

**Fig. 3:**
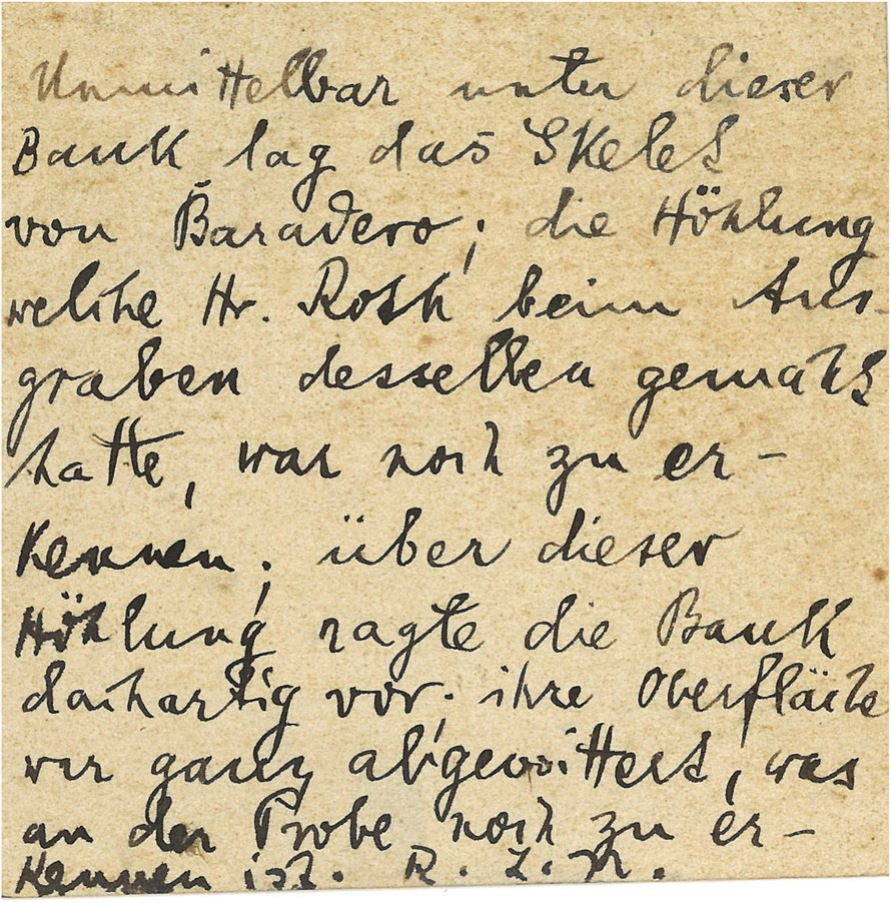
Handwritten note signed by Robert Lehmann-Nitsche that accompanies the Baradero skeleton. Original in German: “Unmittelbar unter diese Bank lag das Skelet von Baradero; die Höhlung welche Hr. Roth beim Ausgraben desselben gemacht hatte, was noch zu erkennen; über dieser Höhlung ragte die Bank dachartig vor; ihre Oberfläche war ganz abgewittert, was an der Probe noch zu erkennen ist. R.L.N “ (Translation in English: Immediately below this layer lays the skeleton of Baradero; the hollow which Mr. Roth had done, while digging it up can still be seen; above this excavation pit the layer projected an outcrop; its surface was completely weathered, which still can be seen in the sample. R.L.N)

**Fig. 4:**
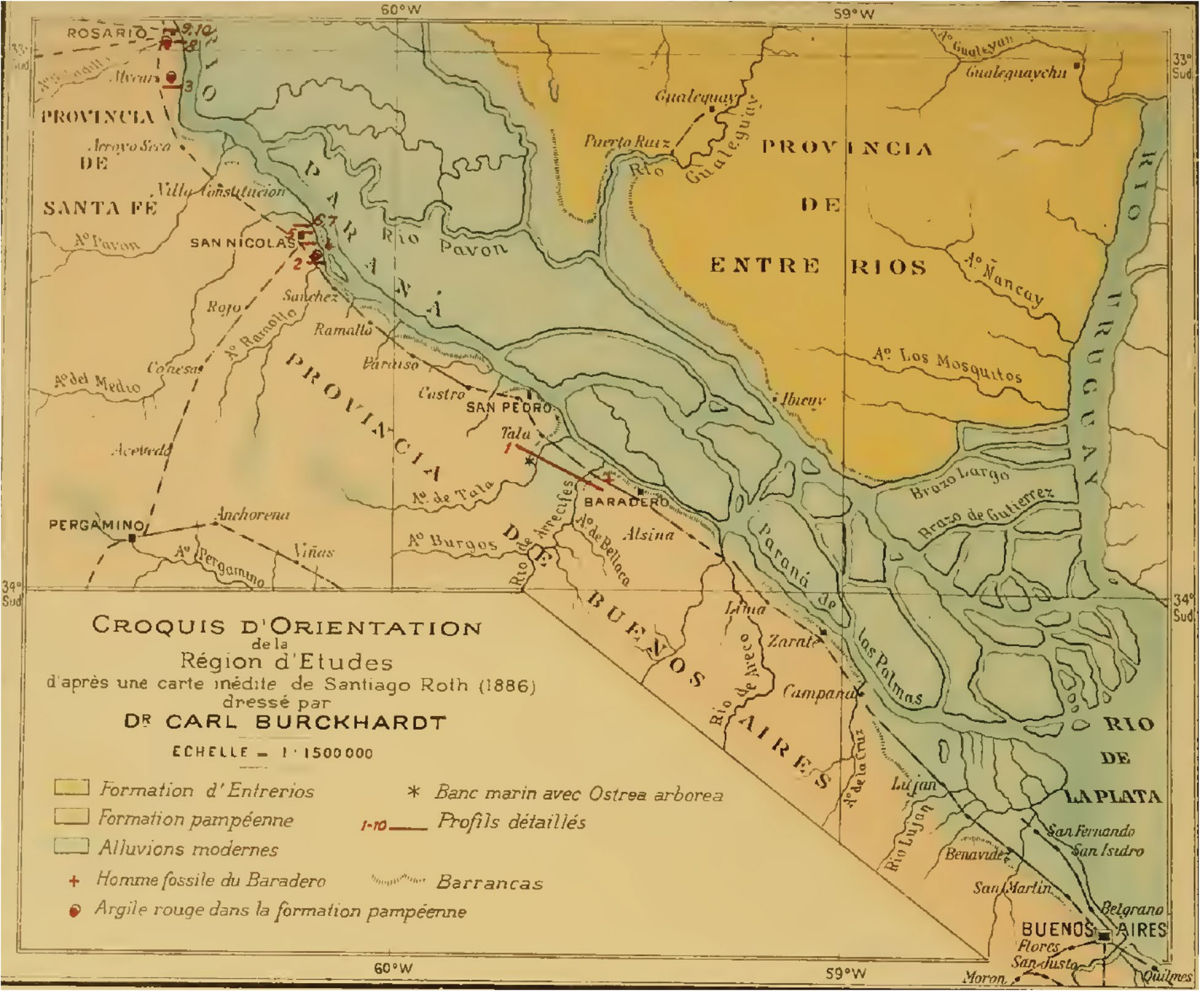
Map created by Carl Burckhardt. The location of the site of the Baradero skull is in the center of the map indicated with a small red cross (Original source: Title “Planche I”; Author Lehmann-Nitsche, 1907; URL: https://publicaciones.fcnym.unlp.edu.ar/rmlp/article/view/1246; License: CC BY 4.0.)

**Fig. 5:**
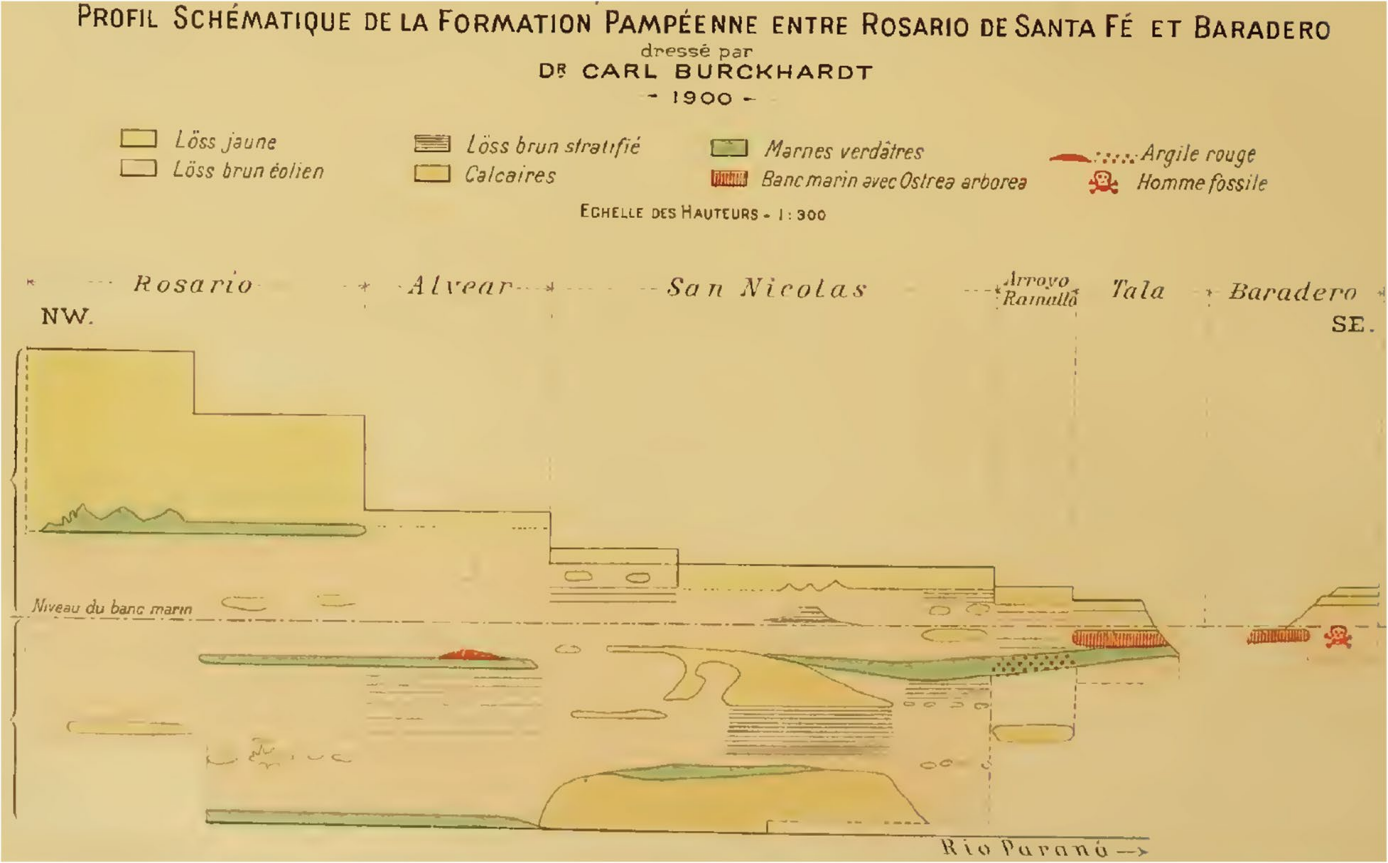
Comparative stratigraphic profiles of the “Pampean Formation” between the cities of Rosario and Baradero created by Carl Burckhardt (at the location of Rosario, Alvear, San Nicolás, Arroyo Ramallo, Tala, Baradero). The stratigraphic layer, where the Baradero skeleton was found is indicated with a red skull (Original source: Title “Planche I”; Author Lehmann-Nitsche, 1907; URL: https://publicaciones.fcnym.unlp.edu.ar/rmlp/article/view/1246; License: CC BY 4.0.)

**Fig. 6:**
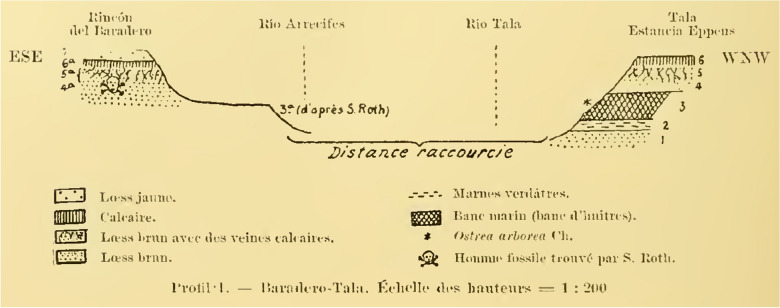
Illustration showing the stratigraphic profile at the site of Baradero in comparison with that of the nearby location Tala (San Pedro). The stratigraphic layer, where the Baradero skeleton was found is indicated with a black skull. This figure was probably prepared by Lehmann-Nitsche based on the profile performed by Carl Burckhardt (see Fig. 1 in this paper). (Original source: Title “Profil 1”; Author Lehmann-Nitsche, 1907:158; URL: https://publicaciones.fcnym.unlp.edu.ar/rmlp/article/view/1246; License: CC BY 4.0.)

In 1899, Lehmann-Nitsche, Roth, and the geologist Carl Burckhardt visited the Parana River ravines, including the archaeological and paleontological sites between Baradero and Rosario cities (Farro, 2008). When visiting the Baradero site, Burckhardt considered that the skeleton has been buried at least one meter below the surface (Dávila, 2022; Lehmann-Nitsche, 1907). In a handwritten note signed by Lehmann-Nitsche, he describes that the excavation pits dug by Roth to extract the skeleton were still recognizable 12 years afterwards (Fig. 3). Carl Burckhardt prepared a map showing the location of the site (Fig. 4) as well as a comparison of stratigraphic profiles showing the geological context of the finding (Fig. 5), which was probably simplified later by Lehmann-Nitsche and used for discussing his interpretations on the antiquity of the finding (Fig. 6). From Fig. 4, we can estimate the geographic coordinates to be −33.79 °S, −59.52 °W. The profile that Burckhardt drew shows that the finding of the Baradero skeleton lies within the eolic brown loess and adjacent to the marine layer that includes oyster shells (*Ostrea arborea*) but not within it (Fig. 5). The scheme provided by Lehmann-Nitsche (Fig. 6) shows a comparison of the stratigraphic profile at the Tala and Rincón del Baradero sites, where the skeleton was found, demonstrating that despite the loess layer in which the Baradero skeleton was found close to the marine layer at Tala, they are not from the same age. As a result, while Roth considered the Baradero skeleton equivalent to the Tertiary age (“Pampeano Intermedio”), Lehmann-Nitsche considered the antiquity of the Baradero skeleton to be Quaternary. There was an extensive debate at the time in relation to the geologic age of the sediments and the fossils found within it—this topic is beyond the scope of this paper and will not be summarized here (Lehmann-Nitsche, 1907; Roth, 1921; Voglino et al., 2023). Moreover, if there was an intentional burial and a pit was excavated for the deposition of the individual, the skeleton would have cut through much older deposits, while the deposition of the skeleton would be more recent. Following today’s standards, it is difficult to arrive at a reliable interpretation of the antiquity of the remains based on the description of the geological deposits. The deposition of the sediments is complex: they have a marine-continental origin, have been reworked by fluvial process, and affected by weathering (Fucks & Deschamps, 2008; Fucks et al., 2011). Only the information on the precise coordinates and depth of the site or the results of radiocarbon analysis would facilitate discussing the antiquity of the human remains. In relation to the cranial shape, the current state of preservation of the skull does not allow us to conduct any morphometric comparative analysis to test the hypothesis on the antiquity of the human remains.

The Baradero skeleton was sold in 1890 by Roth to the École Polytechnique Féderal in Zurich (today the Swiss Federal Institute of Technology in Zurich), as part of a paleontological collection of Pampean fossils, “Catalogue N° 5”, listed under the number 213 (Voglino et al., 2023). It was studied by Martin in 1901, who published his report as part of the extensive monograph by Lehmann-Nitsche (Lehmann-Nitsche, 1907). Although Martin argued that the state of preservation (i.e., the skeleton still being covered by calcareous concretions) prevented any conclusions on its antiquity or biological affinities, he made statements on this regard: (a) some bones present postmortem natural modifications, this being evidence of having been broken while buried, (b) the individual is an adult male and presents a tall stature; and (c) the skeleton presents morphological features similar to those of other contemporary Native Americans. There is also a section written by Ales Hrdlička in his famous monograph “Early Man in South America”, where he interprets and discusses many of the findings and interpretations on the South American human skeletons, although he never observed the Baradero skeleton himself and based his interpretations on the assessments provided in Martin’s publication (Hrdlička, 1912). Hrdlička considered that the Baradero skeleton, as many others in South America, is marked by the scanty geologic record, poor material, and the absence of any conclusive evidence of an ancient age (Hrdlička, 1912). After this, except for some mentions like the one by Castellano (1924), who reused the data by Martin for his own morphometric comparisons, there were no further studies of the skeleton until the present.

In 1914, all the fossils from “Catalogue N°5” were transferred to the new Zoological Museum in the new building of the University of Zurich—now the Department of Paleontology of the University of Zurich, Switzerland, where they are currently housed (Voglino et al., 2023). The collection number of the Baradero skeleton is PIMUZ A/V 4217.

## Osteobiographic assessment of the Baradero individual: comparison of old and new results

### Overall description of the skeleton: body part representation

**Fig. 7:**
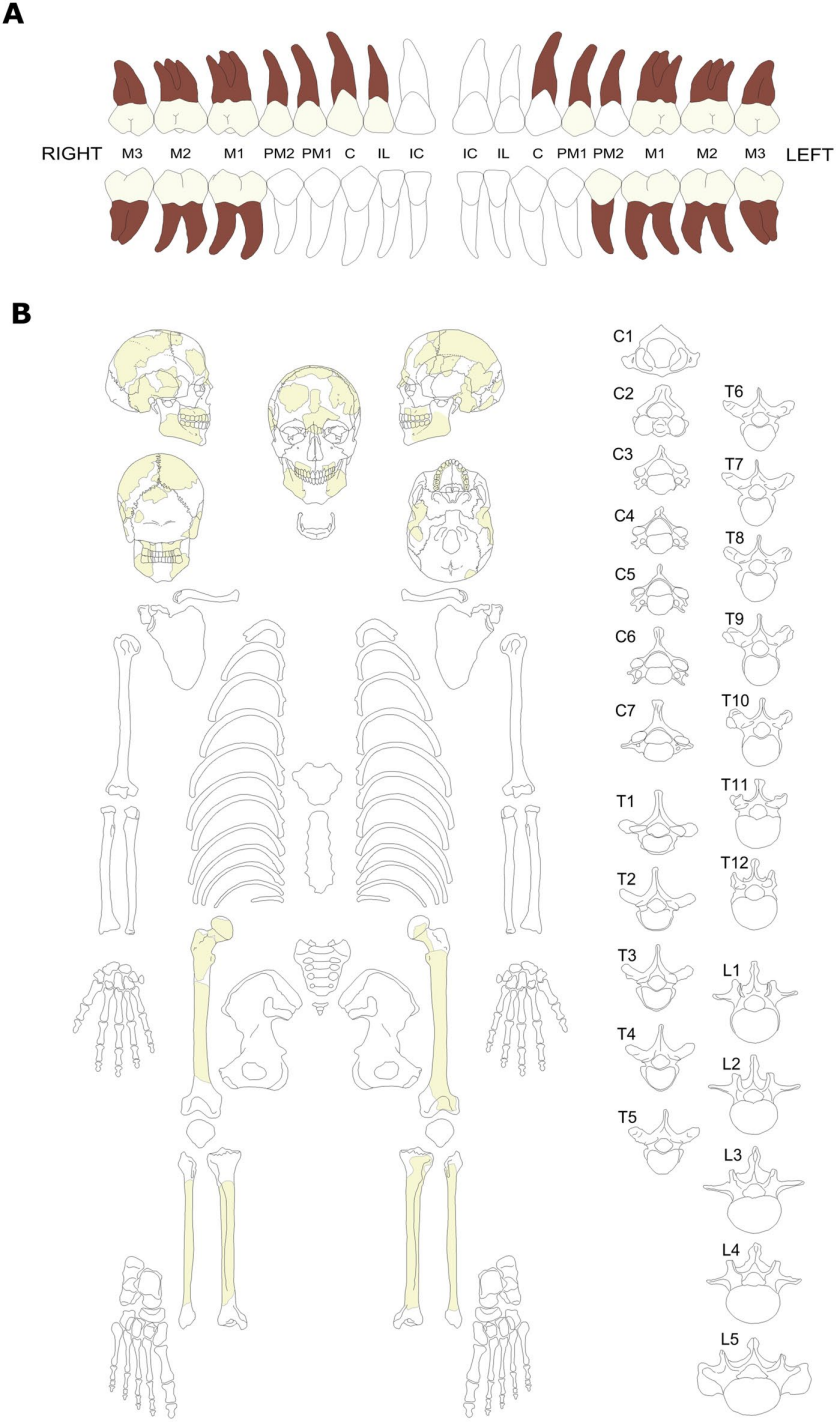
**A** Representation of the dentition of the Baradero individual in the maxilla (upper line) and mandible (lower line). This figure was created based on the modification of the skeletal scheme provided by Langley and collaborators (2016); **B** Schematic representation of the bones from the Baradero individual as of the publication of this article. This figure was created based on the modification of the skeletal schemes provided by Buikstra and Ubelaker (1994) and White and collaborators (2011). The available bones and dental crowns are represented in light yellow, the dental roots in brown

At the moment in which Rudolf Martin studied the skeleton, in 1901, he described fragments of the skull (most of them belonging to the frontal and parietals, but also occipital, right temporal bone, and upper jaw), two parts of the lower jaw, right and left femoral diaphysis, right and left tibia and fibula, and a fragment of the left calcaneus (Martin, 1907). All anatomical elements described by Martin are still present and associated with this individual, except for the calcaneus, which, if present, could be highly fragmented, and difficult to identify with certainty (Fig. 7a–b). There are several smaller and undiagnosable fragments of different sizes and textures. All of the bones are very poorly preserved, the maxilla and mandible probably being the best-preserved elements. According to Roth, the remains were in a better condition before they arrived in Zurich (Martin, 1907: p. 375), meaning that they got broken during the trip. The skull, mandible, and long bone fragments were glued and put together using different materials available at the time, probably with the aim of reconstructing their shape and conducting morphometric analyses. The bones have different coloration: some of the skull bones are darker (e.g., fragments of occipital and parietal bones being dark brown), while the post-cranial elements are characterized by lighter coloration (i.e., yellow to light brown). This could be a sign of a complex taphonomy process in which different parts of the skeleton were exposed to different biological and geological agents. As a result of such poor preservation, in its current state it is not possible to identify any pathology or anatomical anomaly in the cranium or postcranial elements.

**Fig. 8:**
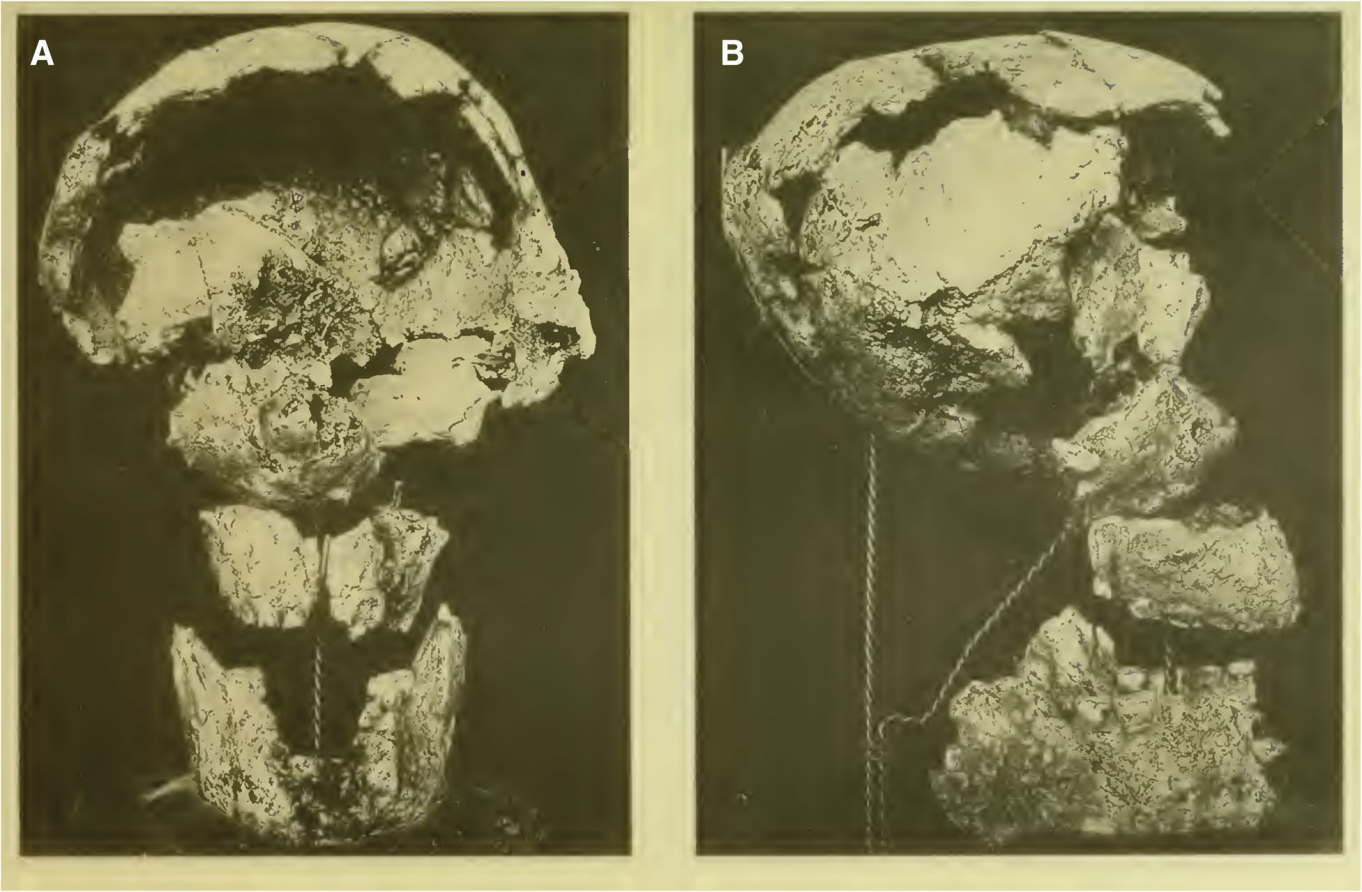
Skull reconstruction of the Baradero skull in frontal (A) and right lateral view (B), probably conducted by Martin in 1901 (Original source: “Planche V”; Author Martin, 1907; URL: https://publicaciones.fcnym.unlp.edu.ar/rmlp/article/view/1246; License: CC BY 4.0.)

In relation to the skull bones, Martin included in his report a photograph of the skull with fragments that had been glued and also put together with wire to create a more representative anatomical reconstruction of the skull shape (Fig. 8). This reconstruction was likely done by Martin himself, after removing the sediment matrix that was keeping the bones together (Fig. 3). However, he mentioned that the skull was prepared by Roth (probably during his reconstructive activities to prepare the fossils that were sent to museums) and also by the technician Mr. A. Dreyer who was instructed by Roth himself (Martin, 1907: p. 375; Voglino et al., 2023). Nowadays, the recognizable skull bones are separated into 19 fragments. From the skull picture presented by Martin, we notice that the skull presents some shape alterations that could probably be interpreted as the result of the post-depositional modifications that he acknowledged in his report (Fig. 8). Those modifications are especially evident posteriorly at the level of the occipital and laterally on the left parietal and temporal bones (Fig. 8). We consider these morphological modifications as having occurred post-mortem as a result of post-depositional processes. We suspect they were not performed during the individual’s life because of the specific location of the morphological alterations (i.e., inferoposteriorly), and the absence of the typical overall changes in the skull shape pattern that characterize the cultural modifications found in southern South America, both intentional and non-intentional (Perez, 2007). Martin described the mandible as being large, curvy, thick, with strong masseter attachment sites. He mentioned that he had only seen similar strong attachments in individuals from Sumidouro cave in Brazil. He took measurements from the thickness at the posterior part and the height of the mandibular body and calculated an index that led him to conclude there are strong similarities between the Baradero mandible and the Neanderthal from Spy cave in Belgium (Martin, 1907: p. 378). We agree that the mandible from Baradero is indeed very robust, but we consider that those morphological similarities with Neanderthals are not revealing of affinities. Due to the artificial reconstruction of the mandible, we are not able to carry out morphometric analysis to compare it with other mandibles from South American individuals, but in general, we can hypothesize that it falls within the range of variation of Native Americans, and from its morphological features we can interpret it as probably belonging to a male (see dedicated section below).

The dentition is also poorly preserved, with most teeth presenting post-depositional fractures and fissures. There is a total of 20 teeth present, including all of the 12 adult molars (Fig. 7a). Most of the anterior teeth are broken or missing together with the anterior part of the mandibular body and the maxilla, which were reconstructed using some artificial material. In addition to the six molars the mandible presents the left second premolar, while the maxilla presents fragments of the right and left first and second premolars, right and left canines, and the right second incisive (Fig. 7a). Martin took two measurements for each molar, which he defined as “length” and “thickness” (probably corresponding to mesiodistal and buccolingual crown diameters) and concluded that the molars from the Baradero individual are quite large (i.e., macrodont), which he considered a typical feature among Native Americans (Martin, 1907). This agrees with the results of global comparative studies of human populations, in which Native Americans have been described as having the largest teeth together with populations from Oceania and Africa (Hanihara & Ishida, 2005). We measured the lower and upper molars and compared our own results with those of Martin and concluded that they are very similar to each other (Table 1). Differences are less than 1.8 mm, being some measurements exactly the same (width of third lower molar), and the most different one 1.8 mm (width of the second lower molar).

**Table 1:** Comparison of the dental measurements taken by Martin and by us (the latter are indicated in brackets). Teeth that today have a poor preservation were not measured

	Right		Left	
	Length	Width	Length	Width
First upper molar	11.5	13	11	13.5
Second upper molar	10 (9.94)	12 (11.98)	10.5 (11.2)	13 (12.09)
Third upper molar	9 (9.03)	12 (10.92)	9 (9.27)	12 (11.8)
First lower molar	12	11	11 (10.67)	10.5 (9.13)
Second lower molar	12 (11.68)	11 (10.84)	12 (12.13)	11.5 (9.72)
Third lower molar	10 (10.22)	11 (11)	9.5 (10.08)	10.5 (10.45)

**Fig. 9:**
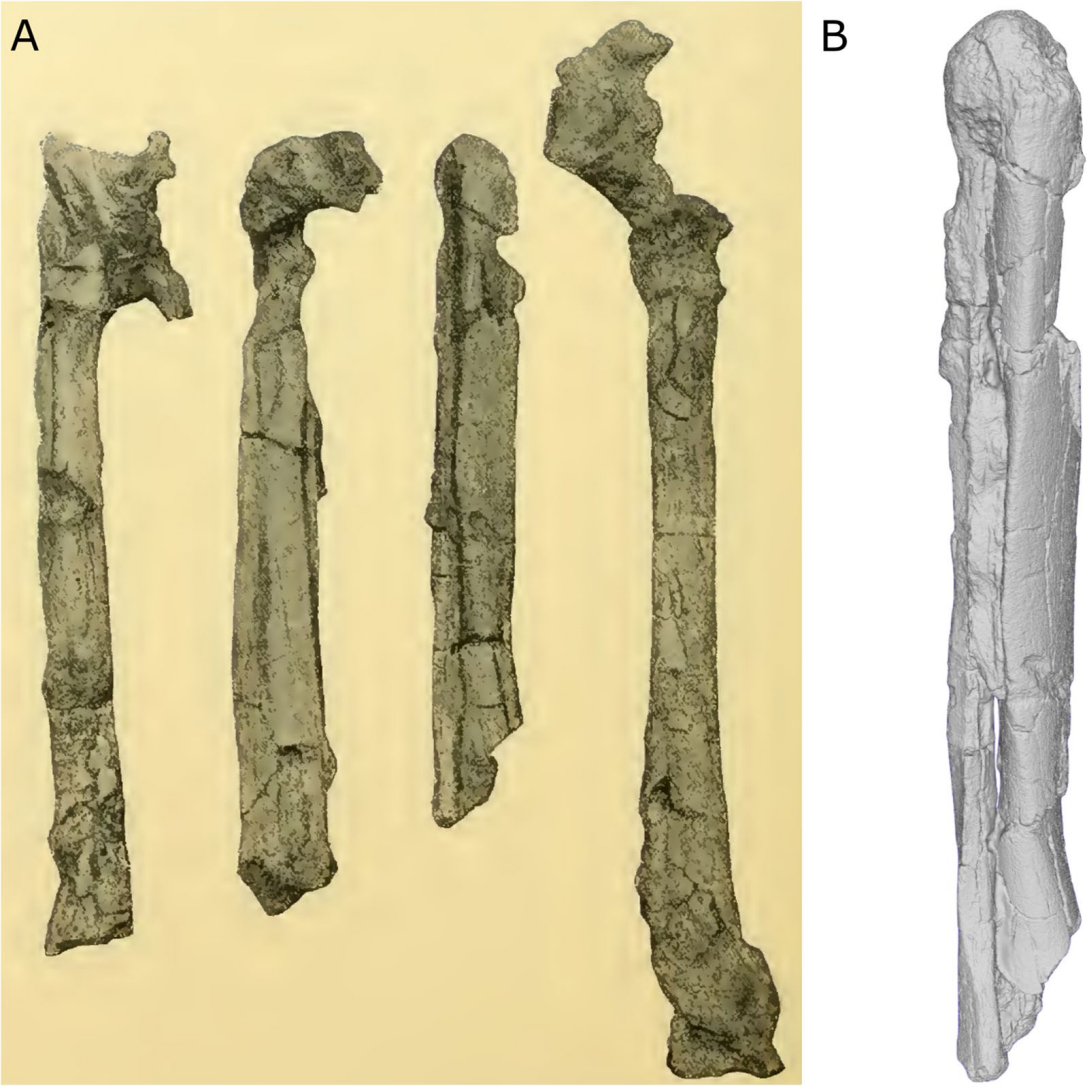
Femura and tibiae from the Baradero skeleton. **A** Picture provided by Martin, probably taken in 1901. From left to right: right femur, left tibia and fibula, right tibia and fibula, left femur. Original source: “Fig. 50”; Author Martin, 1907:382; URL: https://publicaciones.fcnym.unlp.edu.ar/rmlp/article/view/1246; License: CC BY 4.0.); **B** Surface scan showing the right tibia and fibula in their current state

Regarding the postcranial elements, Martin included in his report a description and a photograph of the femora, tibiae and fibulae (Fig. 9). The fibulae are mentioned but not described in detail, probably due to the femur being more diagnostic for the estimation of stature, and the fibula being irrelevant for morphometric assessments at the time. Like the skull bones, the long bones were glued, probably to take measurements. That glue still persists in part today. While this helps in preserving the general shape of the bones and keeping small fragments in place, it does not in fact allow the calculation of any reliable measurements for estimating height or probable sex, since it is difficult to evaluate the orientation of the different fragments and if they have been properly joined. The bones still have a large amount of adherent sediment which is acting as a matrix to keep the fragments together.

The left femur is in more complete condition than the right one (Fig. 9). This was acknowledged by Martin, who described the left femur as being attached to part of the pelvis via the acetabular cavity. While still present, currently the proximal epiphysis has split from the femur diaphysis and the femur head has also been separated. Based on comparisons with other individuals from the collections at the Anthropological Institute of Zurich, Martin performed a reconstruction of the left femur by estimating the missing parts to make some measurements of this bone (Martin, 1907). He then registered the maximal length, and two different antero-posterior diameters of the diaphysis (medial and proximal). Based on the length of the femur (472 mm) he estimated the height of this individual as approximately 170 cm, i.e., taller than the individuals from Fontezuelas (Argentinean Pampas) and Sumidouro cave (Eastern Brazil), as reported by Hansen at the time (Hansen, 1888). Using these measurements, he also calculated indexes and concluded that the Baradero femur presents average values when considering the contemporary and prehistoric variation of Native Americans. Unfortunately, in its current conditions (distal epiphysis missing and split into three parts), we cannot estimate the individual height following current standards.

In his report, Martin described the two tibiae and two fibulae of this individual, although he only mentioned the tibiae in the epigraph of the picture of the long bones (Fig. 9a). Currently, we can identify the two tibiae, as well as both fibulae. While the right fibula is present as a separate bone, the left one is strongly attached to the left tibia consolidated by the sediment matrix (Fig. 9b). They were probably also attached to each other at the time when Martin studied them. Martin calculated antero-posterior diameters for the tibia and concluded they are platymeric (i.e., mediolaterally broad), reaching similar results for the femur. Finally, there are no bones from the thorax, pelvis, arms, hands and feet that could be identified (Fig. 7b).

### Morphological estimation of the biological sex and the age at death

For the estimation of age at death, the conservation of the full dental arches allows us to assign the age of this individual as an adult, given that all 32 adult teeth have fully erupted during it’s life (Fig. 7a). We evaluated the degree of dental wear of the molars using the traditional classification scheme by Brothwell (1981) to approach a more specific age range. Of the nine molars that were available for analysis, two fell into the second stage (17–25 years), five in the third stage (25–35 years), and two in the fourth stage (> 45 years). Thus, we interpret that the individual is likely a middle-aged adult (~ 30–35 years), since some of the teeth show moderate wear, but no teeth show severe dental wear. However, this conclusion should be taken with caution, since dental wear is highly influenced by the hardness of the diet and is not considered as an ultimate diagnostic feature for estimating age, but instead as a complement together with the evaluation of other attributes. Unfortunately, due to poor preservation of the skeleton we were not able to estimate the age based on other skeletal elements such as the degree of skull suture closure. Furthermore, some diagnostic features such as pubic symphysis or ribs were absent, so we were not able to complement the age evaluation of the dental wear with any other information.

The diagnostic features for estimating biological sex are not preserved enough or are still obscured by sediment, so it is not possible to determine sex by evaluating morphological features in this individual with enough confidence. Particularly, the most sexually dimorphic structure, the pelvis, is not present at all, and the second one, the skull, is poorly preserved (Buikstra and Ubelaker, 1994). The dimorphic features of the skull that are diagnostic for estimating sex are either not present (supra-orbital margin, glabella) or poorly preserved (nuchal crest, mastoid process) (Martin & Saller, 1957). The mental eminence of the mandible is also absent, since this part has been replaced with artificial material to reconstruct the shape of its anatomical structure. However, if we take into consideration the overall robusticity of the mandible, maxilla, and long bones, we could agree with Martin’s assessment of this individual being a male (Martin, 1907). These results have been also confirmed by our genetic analysis, showing the chromosomic sex as that of a male, as detailed below.

## Radiocarbon analysis

Dating of the Baradero skeleton was attempted by Toledo, who analyzed fragments of enamel and dentine from the upper second molar and a skull fragment through the Uranium series method (Toledo, 2017). According to Scheinsohn, Toledo obtained minimum ages below 5000 year BP (Scheinsohn, 2021). Considering the limitations of the Uranium series method for studying bone and tooth samples (Bischoff et al., 1988), we aimed at conducting a radiocarbon study. We extracted samples from a petrous bone and a long bone diaphysis fragment for conducting ^14^C analysis. Unfortunately, the poor preservation of the bones did not allow recovering enough collagen for conducting radiocarbon analysis to assess the antiquity of the individual.

## Ancient DNA analysis

To enable attaining viable DNA from the biological tissues available from the Baradero remains, two tissue types were planned for sampling: bone and tooth dentin. Thus, the mandible (bone) and one tooth were sampled with standard protocols for ancient DNA (aDNA) analysis. These efforts only yielded an estimate usable for genetic sex determination, but not enough endogenous DNA data for contextualizing the ancestry of the individual. Here, we report the aDNA protocol used and the results obtained.

### Sample preparation, DNA extraction, sequencing protocols

All materials were sampled in the aDNA Clean Lab associated with the Institute of Evolutionary Medicine at the University of Zurich in Switzerland in 2021. Work in this space is conducted in accordance with well-established protocols for clean lab work, including unidirectional workflows, the use of extensive personal protection equipment, and stringent sterilization procedures with instruments treated with UV light and bleach or DNase solution to avoid modern contamination (as outlined in Fulton & Shapiro, 2019).

First, the mandible with teeth was externally decontaminated using ultraviolet irradiation for 15 min on each side. To obtain sterile tissue samples, two locations were selected from the right mandible: tooth dentin and bone tissue. Inner dentin was sampled from the third molar, which visually appeared the most intact and accessible. Using a handheld drill attached to a NSK EMax EVOlution micromotor (NSK410-S, NSK Nakanishi, Inc. Tochigi, Japan) with sterilized bits, the crown and surrounding alveolar bone of the tooth was carefully ablated with a spherical burr to remove external dirt and glue, then the crown was transversely cut close to the cementoenamel junction with a sterilized sectioning blade. This method exposed the inner dentin/pulp cavity for sampling and left the enamel crown intact for later reconstruction or future analyses. The crown was removed in one piece without damaging the mandible. While the external crown seemed macroscopically intact, the dentin tissue within the crown and pulp cavity was chalky in appearance and texture, with a beige color. Two powder extractions were retrieved from the crown dentin and the root dentin using sterilized spherical burr drills for each collection (Table 2).

**Table 2:** Sample details for the ancient DNA analysis

Lab ID	Sampling material locations	Collected tissue details	Powder collected (mg)
Roth1	Intact third molar	Crown dentine	69.9
Roth2	Intact third molar	Root dentine	54.9
Roth3	Fragmented right mandible, lingual/medial side	Cortical bone tissue	57.1
Roth4	Fragmented right mandible, lingual/medial side	Cortical bone tissue	60.4

Bone tissue was additionally sampled from the same bone fragment. We targeted a section of the mandible fragment corpus (body) on the medial/lingual side, near the inferior-facing border, where the cortical bone is supposedly thicker. A small part of this section’s surface appeared to have less dirt or sealant. A small window of approximately 1.0 × 0.5 cm was removed from the bone surface with a sterilized spherical burr drill bit. Dust was wiped away, followed by a 10 min, superficial decontaminating UV-treatment. The cortical surface appeared macroscopically intact with some weathering, but the sealant gave a false impression of the tissue integrity underneath. The external surface was generously removed to access sterile cortical bone. Its texture was soft and chalky, much like the tooth dentin. While this texture was consistent throughout the inner tooth during sampling, there were some veins of dark discoloration within the bone tissue, possibly indicating contamination of microbial or fungal agents. During sampling, these discolored veins were avoided whenever possible in favor of the whiter cortical tissue for collection. From this window, two samples of bone tissue were collected in duplicate for extraction using a new spherical drill burr (Table 2).

The four powdered samples and one negative extraction blank were demineralized and digested in a buffer (0.45 M EDTA, 0.25 mg/mL Proteinase K, 10% N-lauryl-Sarcosine) with an overnight incubation at 37 °C, with nutation (Dabney, et al., 2013; Dabney & Meyer, 2019). Following this step, DNA was extracted from roughly 1 mL of digested product using silica columns (QIAGEN N.V.; Hilden, Germany). Extracted DNA was converted into double-stranded Illumina libraries with double indexes/barcodes, using established protocols with minor modifications (Kircher, et al., 2012; Meyer & Kircher, 2010). Libraries were later quantified and analyzed for fragment size with a TapeStation System (Agilent Technologies, Santa Clara, CA), then diluted to 10 nM to be pooled equimolarly. We sequenced the libraries in two rounds: first with shotgun sequencing, and then, after verifying the presence of viable aDNA, with whole-genome capture.

For the shotgun sequencing, the samples and blanks were pooled with other ancient samples from separate projects, for a total of 40 samples. Sequencing was performed with Illumina NextSeq500, in Paired End 75 bp cycles Full Mid Output Flowcell, at the facilities of the Functional Genomics Center Zurich (University of Zurich).

For the whole-genome capture, we used an in-solution capture approach based on modified immortalized probe sequences (Gnirke et al., 2009). A panel of 1,237,207 single nucleotide polymorphisms (SNPs) were enriched from the total DNA in the sequencing libraries (Fu et al., 2013, 2015; Mathieson et al., 2015). Briefly, the libraries were reconditioned by further amplification with IS5/IS6 primers and Herculase II Fusion DNA Polymerase to reach a concentration of 200–400 ng/µL as measured on a NanoDrop 8000 spectrophotometer (Thermo Fisher Scientific Inc.; Waltham, MA). Capture was performed on 5.25 μL of each re-conditioned library using that volume for each capture procedure (Fu et al., 2013, 2015). After enrichment, captured library pools were sequenced on the Illumina Hiseq 4000 (Illumina, Inc., San Diego, CA) platform with 75 cycles providing ~ 20 million reads per assay. Sequencing of captured libraries and blanks was performed with Illumina NextSeq500 HO, in Paired End 75 bp cycles Full High Output Flowcell, at the facilities of the Functional Genomics Center Zurich (University of Zurich).

### Quality control and sequence data processing

**Fig. 10:**
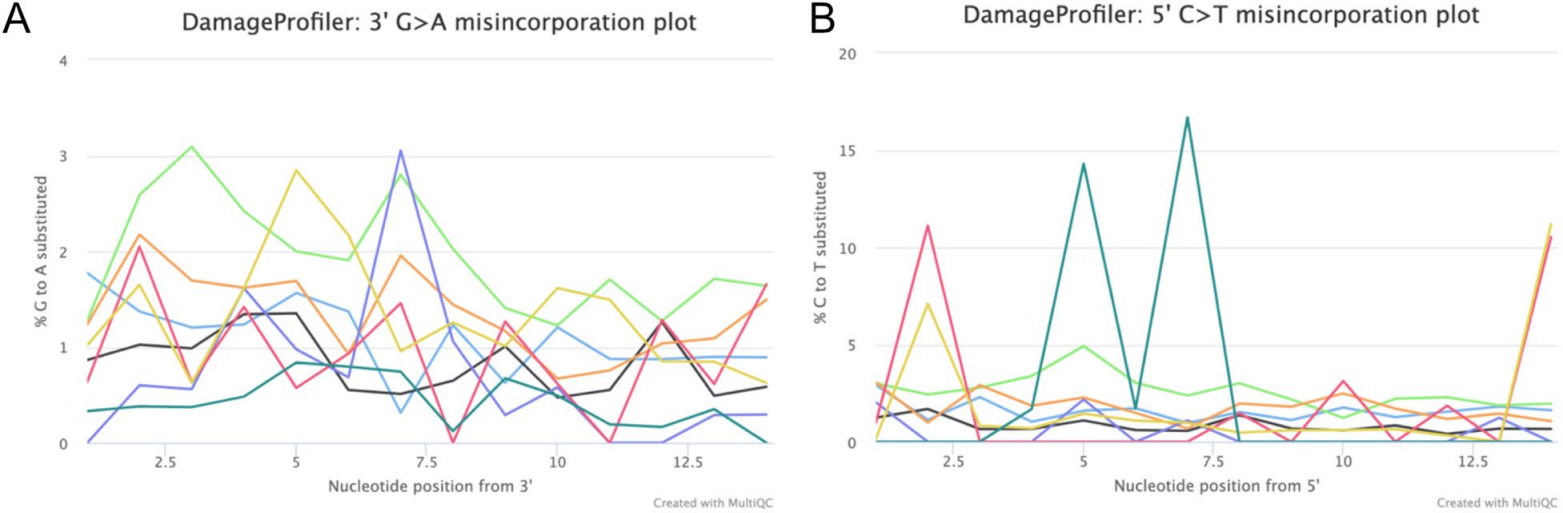
Damage plots for the 3’ (**A**) and 5ʹ ends (**B**) for the four libraries [each line corresponds to a different library and treatment (shotgun or capture)], obtained with the built-in version of DamageProfiler (v0.4.9) for nf-core/eager (v. 2.4.3). The number of reads would be sufficient to clearly distinguish the substitution patterns characteristic for aDNA

For the libraries and blanks, we performed analyses by merging the shotgun and the captured sequence data using nf-core/eager ver. 2.3.4 (Fellows Yates et al., 2021), in an attempt to recover as much genetic information as possible. AdapterRemoval v.2 (Schubert et al., 2016) was used to trim adapter sequences and to remove adapter dimers and low-quality sequence reads (min length = 30; min base quality = 20). The pre-processed sequences were mapped to the human genome assembly GRCh37 (hg19) from the Genome Reference Consortium (International Human Genome Sequencing Consortium, 2001) using the software BWA ver. 0.7.12 (Li & Durbin, 2009) and a seed length of 32. The C to T misincorporation frequencies typical of aDNA were obtained using *mapDamage* 2.0 (Jónsson et al., 2013) to assess the authenticity of the ancient DNA fragments. The misincorporation plots do not allow us to robustly assess the overall authenticity of the aDNA present in the four libraries (Fig. 10). We used samtools mpileup (parameters –*q* 30 –*Q* 30 –*B*) to generate a pileup file from the merged sequence data of all available sequencing data from the four libraries (both shotgun and captured reads) and used a custom script (pileupCaller ver. 8.2.2; Schiffels, 2018) to genotype the resulting data, using a pseudo-haploid random draw approach. For each position on our capture panel, a random read was drawn for each individual and the allele of that read was assumed to be the homozygous genotype of the individual at that position. 271 SNPs passed this filtering procedure.

**Fig. 11:**
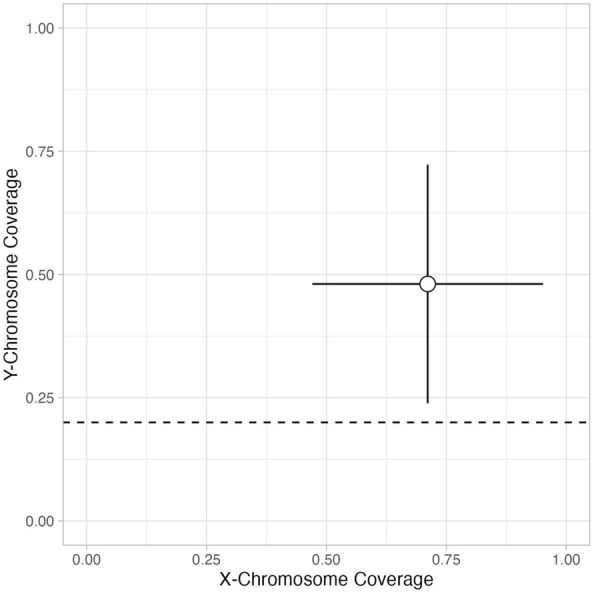
Sex determination based on the SNP coverage for the X and Y chromosomes for the merged sequenced data. Value is plotted ± standard error (SE) for accuracy on the sex determination. Values ± SE above 0.20 on the *y* axis are considered as genetically male

With this low number of SNPs recovered from both the shotgun and capture sequence data we could not perform any downstream population genetics analyses, except evaluating the genetic sex of the individual. The genetic sex was assigned by calculating the ratio and standard error of average X chromosomal and Y chromosomal coverage to the average autosomal coverage at the targeted SNPs (Lamnidis et al., 2018). For assigning sex, an X rate between 0.35 and 0.55 and a Y rate between 0.4 and 0.7 are assigned as male. The genetic sex of the individual appears in the range of male values (Fig. 11). ANGSD was then used to estimate nuclear contamination, since males are expected to be homozygous at each X chromosome position (Korneliussen et al., 2014), but the data retrieved were not sufficient to perform this evaluation.

## Virtual reconstruction of the Baradero skull

**Fig. 12:**
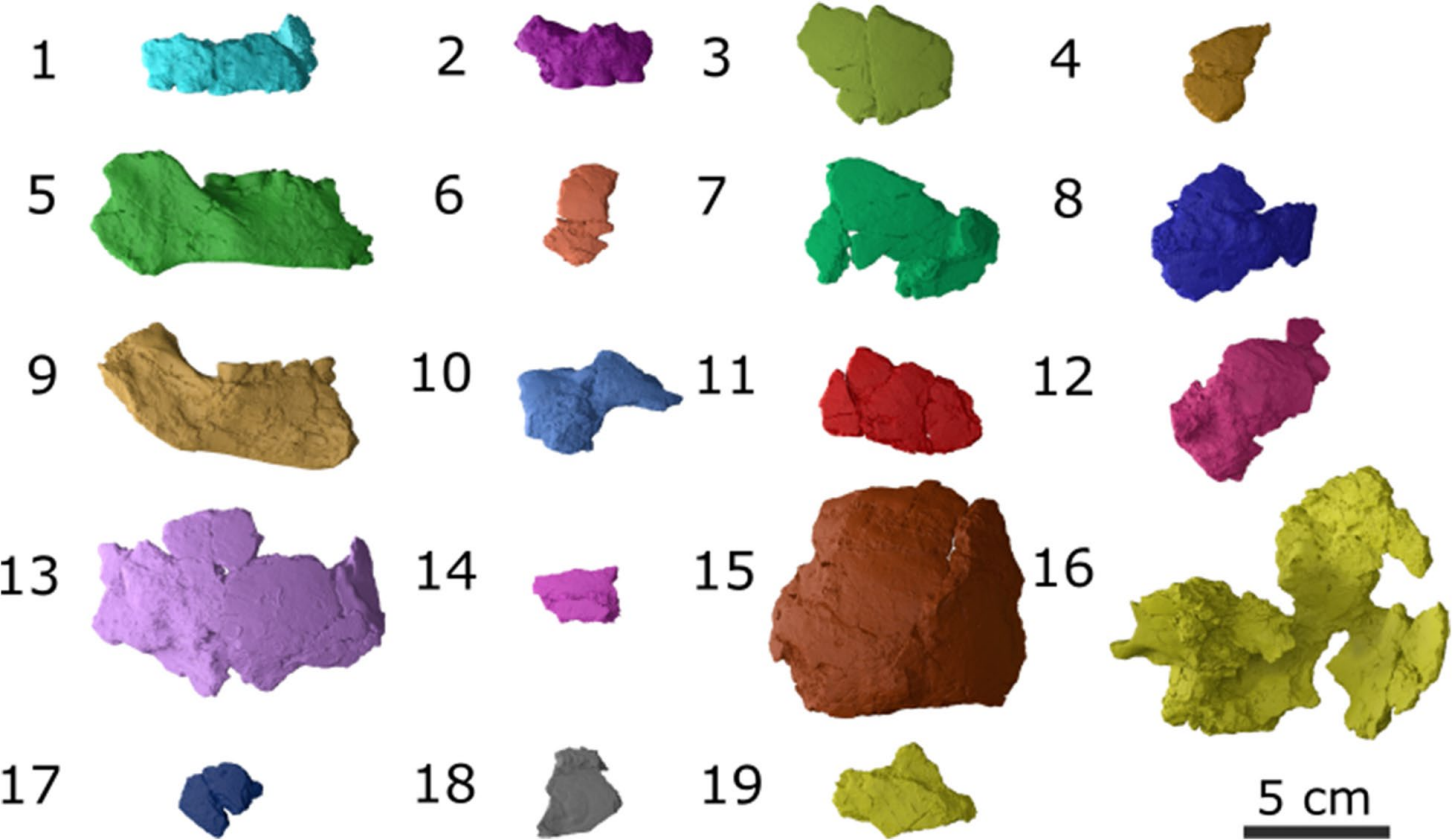
3D models produced from the CT-scans of the 19 skull bone fragments. See Table 2 for references on which bones are contained in each fragment. Since fragments 17 and 18 did not present any diagnostic features, it was not possible to identify them and were not employed in the reconstruction

A total of 19 skull fragments were micro-CT-scanned during a single scanning session using the high-resolution computer tomography Nikon X TH 2255 T, at the Departments of Paleontology and Anthropology at the University of Zurich (Fig. 12). The following parameters were selected for scanning the skull fragments: voxel size 12 μm, Xray kV 180, Xray uA 298, and copper filter with a thickness of 1 mm was used. With the CT-scan files obtained, two of us (TS, IGLC) conducted two cranial reconstructions that were supervised by LPM. The cranial reconstructions were performed using different softwares: Avizo 2019.1 (Thermo-Fisher Scientific, 2019) Blender v.3.5 (Community, 2018) and Slicer Morph v.4.10.2 (Rolfe et al., 2021). The procedure was the same in both cases. We cropped the fragments from the main stock of images. Then, we followed the protocol by Menéndez et al. (2019), first creating meshes that were placed following an anterior to posterior sequence on top of the model of a skull that was used as a template. Diagnostic anatomical features as well as thickness, breakage patterns, and matching edges were used as criteria for placing and orienting the skull fragments. Access to the actual bone fragments was of help for evaluating diagnostic criteria and comparing the matching edges in the virtual space. As a template, we used the skull and the mandible of a male individual from north Patagonia, which helped as a guide and anatomical reference. By making the template transparent we situated it in the background and proceeded to place the fragments of the Baradero skull on top of it by following the anatomical orientation of the bones provided by the skull template.

**Fig. 13:**
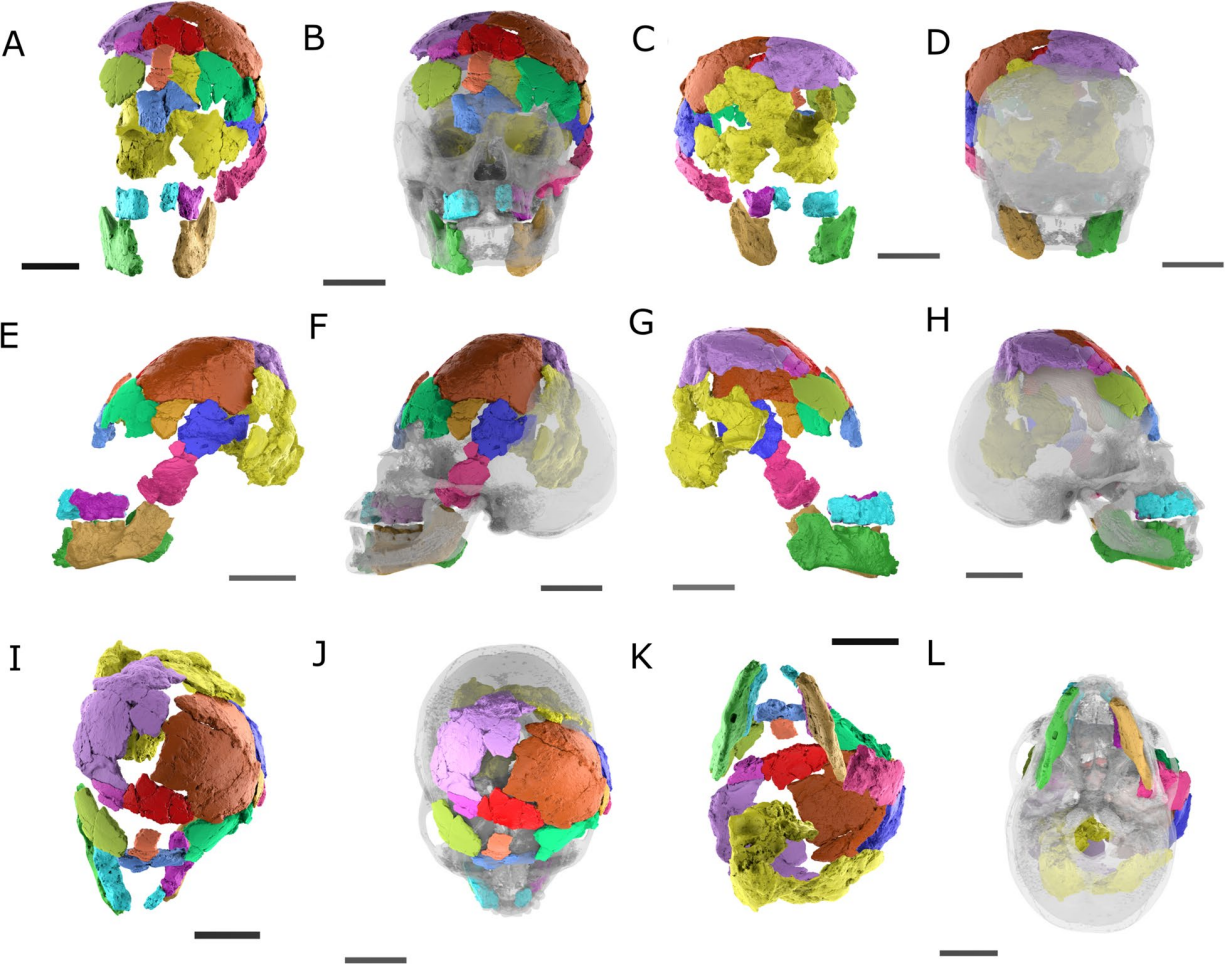
Virtual reconstruction of the skull presented from different views: **A** frontal; **B** frontal with template; **C** posterior; **D** posterior with template; **E** left lateral; **F** right lateral with template; **G** right lateral; **H** right lateral with template; **I** superior; **J** superior with template; **K** ventral; **L** ventral with template. The template is displayed in transparency as a reference of the skull shape without modifications. The scales indicate 5 cm in every case

The consensus and final shape that was obtained by combining the two independent reconstructions is shown in the different views presented in Fig. 13. The 3D surface of the cranial reconstruction is available at the online repository MorphoMuseuM (Lebrun & Orliac, 2016) under the identifier M3#1198 (Menéndez et al., 2023). As it can be observed from Figs. 8, 12, and 13, the main challenges faced during this reconstruction were the difficulties in identifying diagnostic features in the fragments due to the poor preservation of the bones, as well the existence of post-depositional modifications that not only altered the shape of the bones and resulted in some fragments do not necessarily matching each other (Table 3). Fragments belonging to the occipital and parietals were especially affected and present alternations in their shape (Fig. 12: fragments 13, 15, 16). As a result of the post-depositional alterations and the incompleteness, the skull reconstruction should be taken as an approximation of the original skull shape, and unfortunately, cannot be used for registering landmarks or measurements for further comparative craniometric analysis.

**Table 3:** Identification of skull fragments

Bone	Reference in Fig. 13	Preservation
Frontal	3, 6, 7, 10, 11, 14	Good
Maxilla	1, 2	Glued
Mandible	5, 9	Very good
Left parietal	4, 8, 11, 15	Post-depositional alterations
Right parietal	13,	Post-depositional alterations
Left temporal	12	Post-depositional alterations
Right temporal	16	Post-depositional alterations
Occipital	16	Post-depositional alterations
Sphenoid	7	Good
Unidentified fragments	17, 18	N/A

## Final considerations

The study of the Baradero skeleton represents an example of the contextualization of historical material even when preservation conditions are poor. This is especially—but not exclusively—the case for those ones that are ancient, and, therefore, have a high historical value. The preservation conditions are the result not only of the environmental conditions at the site and region, where the individuals were buried and are found, but also of the time that has passed since the death of the individual: the longer the time passed since the burial, the more the body has been exposed to natural and geological agents that contribute to its decay (Knüsel & Robb, 2016). Examples of this are the skull series from *Serra da Capivara* in Brazil, for which collagen preservation is poor (Menéndez et al., 2022), and the skeletal series of Arroyo Seco 2 in Argentina and OGSE80 in Ecuador, in which most cranial parts are kept together by sediment matrix and in most cases present post-depositional modifications (Politis et al., 2012; Ubelaker, 1980). As a result of the former, when collagen is not preserved, researchers are unable to retrieve biomolecular data such as that used for radiocarbon dating and genetic analysis (Korlevic et al., 2018; Kontopoulos et al., 2020). Unfortunately, there are no alternatives for conducting genetic and radiocarbon analyses when biomolecular data are not preserved, although a sustainable practice would be testing the content of collagen before sampling by using non-destructive bone collagen prescreening techniques, such as near-infrared spectroscopy (Sponheimer et al., 2019). As for post-depositional alterations and fragmentation, recent work has focused on developing methods and protocols for reconstructing anatomical structures, such as skulls. Virtually reconstructed skulls are informative on multiple aspects and could be used for building an osteobiographic profile and/or studying the biological variation of individuals from the Americas (Davis et al., 2021; Menéndez et al., 2019).

Two challenges we faced in this project were the highly fragmented nature of the skeleton, with poor preservation, and post-depositional alterations. We provide alternatives for dealing with these issues. The results of this study show that an interdisciplinary approach including morphological and biomolecular analyses, applied to poorly preserved skeletons, contributes to the investigation of ancient individuals, as it further advances a better understanding of specimens found in historical collections. These analyses can shed on some aspects of the debate, such as the assessment of sex, age, and the antiquity of individuals that have been at the center of local and international discussions (e.g., Gordón et al., 2013; Politis & Bonomo, 2011). Future studies on the Baradero skeleton could focus on optimizing the analysis of available anatomical structures, e.g., using the bony labyrinth of the inner ear to evaluate biological affinities to other early Holocene individuals, or implementing non-destructive bone collagen prescreening techniques such as near-infrared spectroscopy to search for anatomical parts that may be useful for conducting further biomolecular analysis, and even looking into the proportional size of the pulp chamber to have an approximation to age-at-death, if corrections due to dental wear could be implemented.

## Data Availability

The samples are available at the Department of Paleontology of the University of Zurich. The 3D virtual reconstruction of the skull is published (Menéndez et al., 2023) and available at the online repository MorphoMuseuM under the number M3#1198 (https://morphomuseum.com/). For the ancient DNA analysis, the set of 271 SNPs retrieved for genetic sex determination is available upon request to the authors.
